# Pre-treatment amygdala activation and habituation predict symptom change in post-traumatic stress disorder

**DOI:** 10.3389/fnbeh.2023.1198244

**Published:** 2023-07-10

**Authors:** Cecilia A. Hinojosa, Michael B. VanElzakker, Navneet Kaur, Julia M. Felicione, Meredith E. Charney, Eric Bui, Luana Marques, Paul Summergrad, Scott L. Rauch, Naomi M. Simon, Lisa M. Shin

**Affiliations:** ^1^Department of Psychiatry and Behavioral Sciences, Emory University School of Medicine, Atlanta, GA, United States; ^2^Department of Psychiatry, Massachusetts General Hospital and Harvard Medical School, Boston, MA, United States; ^3^Department of Cancer Biology, Wake Forest School of Medicine, Winston-Salem, NC, United States; ^4^Department of Psychology, Tufts University, Medford, MA, United States; ^5^Normandie Univ, University of Caen Normandy (UNICAEN), L'Institut national de la santé et de la recherche médicale (INSERM), U1237, PhIND “Physiopathology and Imaging of Neurological Disorders”, NEUROPRESAGE Team, (Institut Blood and Brain @ Caen-Normandie), GIP Cyceron, Caen, France; ^6^Centre Hospitalier Universitaire Caen Normandie, Caen, France; ^7^Department of Psychiatry, Massachusetts General Hospital, Center for Anxiety and Traumatic Stress Disorders, Boston, MA, United States; ^8^Department of Psychiatry, Tufts University School of Medicine, Boston, MA, United States; ^9^Institute for Technology in Psychiatry, McLean Hospital, Belmont, MA, United States; ^10^Department of Psychiatry, McLean Hospital, Belmont, MA, United States; ^11^Department of Psychiatry, New York University (NYU) Grossman School of Medicine, New York, NY, United States

**Keywords:** post-traumatic stress disorder, prolonged exposure therapy, fMRI, biomarkers, symptom change

## Abstract

Trauma-focused psychotherapy approaches are the first-line treatment option for post-traumatic stress disorder (PTSD); however, up to a third of patients remain symptomatic even after completion of the treatment. Predicting which patients will respond to a given treatment option would support personalized treatments and improve the efficiency of healthcare systems. Although previous neuroimaging studies have examined possible pre-treatment predictors of response to treatment, the findings have been somewhat inconsistent, and no other study has examined habituation to stimuli as a predictor. In this study, 16 treatment-seeking adults (M_Age_ = 43.63, *n* = 10 women) with a primary diagnosis of PTSD passively viewed pictures of emotional facial expressions during functional magnetic resonance imaging (fMRI). After scanning, participants rated facial expressions on both valence and arousal. Participants then completed eight weekly sessions of prolonged exposure (PE) therapy. PTSD symptom severity was measured before and after treatment. Overall, participants showed symptomatic improvement with PE. Consistent with hypotheses, lesser activation in the amygdala and greater activation in the ventromedial prefrontal cortex during the presentation of fearful vs. happy facial expressions, as well as a greater decline in amygdala activation across blocks of fearful facial expressions at baseline, were associated with greater reduction of PTSD symptoms. Given that the repeated presentation of emotional material underlies PE, changes in brain responses with repeated stimulus presentations warrant further studies as potential predictors of response to exposure therapies.

## 1. Introduction

Trauma-focused psychotherapy is the leading treatment option for individuals with post-traumatic stress disorder (PTSD) (Watkins et al., 2018); however, up to a third of patients who complete such treatment remain symptomatic (Bradley et al., 2005). Previous studies have examined whether pre-treatment demographic, clinical, or cognitive variables in those with PTSD can predict treatment response. For example, individuals who are female, experience lesser pre-treatment numbing symptoms, and have higher scores on verbal memory tests exhibit better responses to trauma-focused psychotherapy (Tarrier et al., 2000; Karatzias et al., 2007; Nijdam et al., 2015). Ideally, this type of information could be used by clinicians to select the most optimal treatment for individual clients, thereby expediting the improvement in symptoms. More recently, biological measures have been found to be more accurate than demographic and clinical variables in predicting treatment response in anxiety disorders (Ball et al., 2014).

Neuroimaging studies have shown that patients with PTSD who exhibited greater pre-treatment activation in the amygdala (Bryant et al., 2008, 2021; van Rooij et al., 2016; Fonzo et al., 2017) and insula (van Rooij et al., 2016) during the presentation of emotionally negative stimuli had a poorer response to treatment. In contrast, participants with greater activation in the ventrolateral prefrontal cortex, dorsal striatum, medial prefrontal cortex (mPFC) (Falconer et al., 2013; Duval et al., 2020), inferior parietal lobe (van Rooij et al., 2015), and greater functional connectivity within the ventral attention network (e.g., insula, dorsal anterior cingulate, anterior middle frontal gyrus, and supramarginal gyrus) (Etkin et al., 2019) had a better response to treatment. The role of the anterior cingulate cortex (ACC) in predicting treatment response remains unclear, given that the findings in this region have been inconsistent across studies. For example, greater pre-treatment activation in the rostral ACC (rACC) has been shown to predict both better (Felmingham et al., 2007) and poorer responses to treatment (Bryant et al., 2008). Likewise, greater pre-treatment activation in the dorsal ACC (dACC) has been shown to predict both better (Aupperle et al., 2013; Fonzo et al., 2017) and poorer (Aupperle et al., 2013; van Rooij et al., 2016; Kennis et al., 2017) responses to treatment.

Most previous studies using pre-treatment neuroimaging to predict treatment response in PTSD have used brain activation measures as the predictors of outcome. However, other measures may be useful, such as the *change* in activation or fMRI blood oxygenation level-dependent (BOLD) signal over repeated stimulus presentations (e.g., Wright et al., 2001; Fischer et al., 2003; Protopopescu et al., 2005). Indeed, such changes in amygdala and ACC function may reflect a capacity for the extinction of conditioned fear (Myers and Davis, 2007), a process that is akin to that of exposure therapy. Given that the amygdala is involved in the acquisition and expression of conditioned fear and the rACC and surrounding ventromedial prefrontal cortex (vmPFC) are involved in retaining memories of extinction (reviewed in VanElzakker et al., 2014), one might hypothesize that a greater pre-treatment decline in BOLD signal in the amygdala and a lesser decline (or an increase) in BOLD signal in the rACC/vmPFC over repeated stimulus presentations ought to predict greater subsequent improvement with exposure therapy.

Although the main goal of this study was to uncover potential biomarkers associated with symptom reduction, in *post hoc* exploratory analyses, we also wanted to determine whether less costly and less burdensome behavioral measures (i.e., subjective ratings of the facial expression stimuli) are associated with symptom reduction. Additionally, given the relatively high dropout rates associated with PE, exploratory analyses also assessed whether pre-treatment brain activation was associated with participants' dropout status.

In this study, we assessed (1) the activation in the amygdala and rACC/vmPFC in response to fearful vs. happy facial expressions and (2) the changes in the BOLD signal over repeated presentations of fearful facial expressions to determine whether these measures predict symptomatic improvement after prolonged exposure therapy (PE), a first-line evidence-based treatment that lacks extensive research on biological measures of response (though also see Helpman et al., 2016; Duval et al., 2020; Sheynin et al., 2020). We predicted that (1) lesser activation in the amygdala and greater activation in the rACC/vmPFC in response to fearful vs. happy facial expressions would be associated with greater symptomatic improvement with PE and (2) a greater decline in the BOLD signal in response to repeated presentations of fearful faces in the amygdala and a lesser decline (or an increase) in the rACC/vmPFC would be associated with greater improvement.

Exploratory analyses were conducted to determine whether (1) participants' arousal and valence ratings of fearful and happy facial expressions were related to symptomatic improvement and (2) pre-treatment brain activation differed between participants who prematurely dropped out of the study compared to those who completed the study.

## 2. Materials and methods

### 2.1. Participants

In total, 24 treatment-seeking participants with PTSD (*n* = 14 women) without a history of head injury, neurological disorders, or other major medical conditions were enrolled in the study. Eight participants dropped out before the endpoint and thus had no post-treatment evaluation. Reasons for dropping out included a busy schedule (*n* = 2), no longer wanting to receive PE (*n* = 5), and military deployment (*n* = 1). For exploratory analyses that compared completers vs. dropouts, we chose to exclude the participant who dropped out of treatment due to an unforeseen military deployment. Those who completed treatment reported a greater number of years of education than those who dropped out, *t*(21) = 2.30, *p* = 0.03; no other significant differences in demographic and clinical characteristics were found (*p*s > 0.40; see Supplementary Table 1).

The final sample consisted of 16 participants (*M*_Age_ = 43.63, *SD*_Age_ = 12.57, *n* = 10 women) with PTSD. The inclusion criteria consisted of being an outpatient, at least 18 years of age, with a primary psychiatric diagnosis of PTSD, as defined by the Diagnostic and Statistical Manual of Mental Disorders (DSM-IV) criteria, and a willingness to receive PE as part of this protocol at the Center for Anxiety and Traumatic Stress Disorders. The exclusion criteria included pregnancy as confirmed by an early detection urine pregnancy test taken before fMRI scanning procedures, having a serious medical illness or instability for which hospitalization was likely during the study, having a diagnosis of psychosis, undergoing current compensation or legal action related to the effects of the trauma, having an ongoing relationship with their assailant, contradictions to MRI, and prior intolerance or failure of response to PE. Psychiatric medication was permitted as long as the dose remained constant throughout the study. A total of 7 out of the 16 participants took a stable dose of psychiatric medication, including antidepressants (*n* = 1), benzodiazepines (*n* = 1), anticonvulsants (*n* = 1), or a combination (*n* = 4) (see Table 1 for demographic and clinical characteristics). The Partners Healthcare System (Boston, MA) Institutional Review Board approved this study. Written informed consent was obtained prior to participation.

**Table 1:** Demographic and clinical characteristics of 16 adults with PTSD receiving eight sessions of prolonged exposure.

	**Pre-treatment**	**Post-treatment**	**Change score**	**Percent improvement score**
**Measure**	**Mean (SD)**	**Mean (SD)**	**Mean (SD)**	**Mean (SD)**
Age	43.63 (12.57)			
Education (years)	16.25 (2.59)			
Time since trauma (years)^a^	9.12 (11.20)			
SPRINT score	21.38 (5.76)	11.69 (10.16)	9.69 (7.94)	49.23% (37.62%)
CGI-S score	4.75 (0.77)	3.19 (1.33)	1.56 (1.09)	33.54% (20.73%)
CGI-I score		2.44 (1.15)		

Data given as mean (standard deviation). PTSD, post-traumatic stress disorder; SPRINT, Short Post-traumatic Stress Disorder Rating Interview; CGI-S, Clinical Global Impressions Severity Scale; CGI-I, Clinical Global Impressions Improvement Scale. *n* = 16 unless specified elsewhere.

^a^*n* = 14.

### 2.2. Clinical assessments

Participants were screened and offered study inclusion if all entry criteria were met. Before beginning imaging procedures, participants completed a clinical evaluation, which included: (1) Mini-International Neuropsychiatric Interview (MINI) (Sheehan et al., 1998) to diagnose DSM-IV psychiatric disorders; (DSM-IV) psychiatric disorders; (2) the short PTSD rating interview (SPRINT) (Connor and Davidson, 2001), an eight-item clinician-administered scale, that measures the severity of the core symptoms of PTSD (DSM-IV). To create a SPRINT change score, post-treatment SPRINT scores were subtracted from participants' pre-treatment SPRINT scores. Additionally, this difference was divided by pre-treatment SPRINT scores and multiplied by 100 to yield a SPRINT percent improvement score; (3) the Clinical Global Impressions Severity Scale was used (CGI-S) (Guy, 2000), which is a well-validated single-item clinician-rated scale that measures overall illness severity. To determine symptomatic improvement, post-treatment CGI-S scores were subtracted from participants' pre-treatment CGI-S scores to yield a CGI-S change score. Additionally, this difference was divided by pre-treatment CGI-S scores and multiplied by 100 to yield a CGI-S percent improvement score (which considers baseline CGI-S scores). Furthermore, during the last treatment session, the Clinical Global Impression Improvement Scale (CGI-I) (Guy, 2000), a one-item clinician-administered seven-point scale, that measures the overall improvement of symptoms from the baseline treatment visit to the last treatment visit was used. Specifically, the one item states that “Compared to the patient's admission to the project, this patient's condition is 1 = very much improved since the initiation of treatment; 2 = much improved; 3 = minimally improved; 4 = no change from the baseline; 5 = minimally worse; 6 = much worse; 7 = very much worse since the initiation of treatment.” The results reported herein will focus on SPRINT percent improvement scores as our main *a priori* symptom-related outcome measure, given that it controls pre-treatment SPRINT scores, and there is more variability in the scores compared to the CGI measures. However, if another symptom-related outcome measure was significantly associated with brain activation, the finding was reported for completeness.

### 2.3. Task procedures

In the scanner, each participant viewed grayscale images of six fearful (F), six happy (H), and six neutral (N) facial expressions from a well-validated set (Ekman and Friesen, 1976) displayed in a block design using MacStim 3.0 software. Each type of facial expression was posed by three men and three women. Each facial expression was presented for 200 ms, with a 300-ms interstimulus interval. Within blocks, 56 presentations of facial expressions were presented in a pseudorandom order such that expressions of a single individual were never presented twice in a row. Facial expressions were presented in separate alternating blocks per functional run (e.g., +N F H F H F H N+), with each block lasting for 28 s, and participants were scanned during three functional runs. Previous studies have shown amygdala habituation across runs (Breiter et al., 1996; Whalen et al., 1998; Wright et al., 2001; Fischer et al., 2003); therefore, we decided *a priori* to test our hypotheses in the first run only. The order of emotional expression blocks was counterbalanced across participants.

After exiting the scanner, participants rated the facial expressions on 7-point valence and arousal scales. Specifically, valence was measured on a scale from −3 to 3, with −3 representing the most negative and 3 representing the most positive valence. Arousal was measured on a scale from 0 to 6, with 0 representing the lowest and 6 representing the highest. We assessed correlations between participants' average stimulus ratings and SPRINT percent improvement scores.

### 2.4. fMRI procedures

Functional MRI data were collected using a Symphony/Sonata 1.5-Tesla whole-body high-speed imaging device equipped for echo planar imaging (Siemens Medical Systems, Iselin, NJ) with a 12-axis gradient head coil. We restricted head movement by using expandable foam cushions. After acquiring an automated scout image, field homogeneity was optimized by conducting shimming procedures. A high-resolution 3D magnetization-prepared rapid acquisition gradient echo (MPRAGE) sequence was gathered in a sagittal plane, with scan parameters as follows: repetition time/echo time/flip angle as 2.73 s/3.39 ms/7°, respectively. Gradient echo functional images were gathered in 24 coronal slices angled perpendicular to the anterior commissure-posterior commissure line with slice thickness at 7 mm, skip 1 mm; voxel size, 3.1 x 3.1 x 7 mm, and with the following parameters: repetition time/echo time/flip angle as 2.8 s/40 ms/90°, respectively.

### 2.5. Treatment procedures

After scanning was complete, participants underwent eight weekly 90-min treatment sessions of PE with a doctoral-level clinician. Eight PE sessions have been shown to be an adequate dose in both clinical (US Department of Veterans Affairs., 2012; Karlin and Cross, 2014) and research settings (Maguen et al., 2019; Valenstein-Mah et al., 2019; Shiner et al., 2020; Sripada et al., 2020). Treatment sessions consisted of three components: (1) educating the patient on how to process their emotional memory, (2) effectively exposing the patient to reminders of their trauma *via* imaginal exposure during treatment sessions, and *in vivo* exposure as homework long enough to allow any negative emotional reaction to decline, helping the patient to restructure their disordered thinking of the traumatic event, and (3) reviewing what was learned during exposure sessions (Foa et al., 2007).

### 2.6. Data analysis

#### 2.6.1. Functional MRI

Image preprocessing and statistical analyses were performed using SPM8, a statistical parametric mapping software package to perform the functional region of interest (ROI) based analyses (http://www.fil.ion.ucl.ac.uk/spm/; Wellcome Department of Imaging Neuroscience, London, UK). Functional images of each participant were co-registered to their high-resolution structural MRI image, smoothed (4 mm), and spatially normalized with standard stereotactic space (Montreal Neurological Institute, MNI). Hypotheses were tested as contrasts in which linear compounds of the model parameters were evaluated using the t-statistics, which were then transformed into z-scores. We computed contrasts in each participant (the first level) and then used those contrasts in group analyses (the second level). The first-level contrasts computed in each participant were: fearful vs. happy (all 3 blocks of fearful versus all 3 blocks of happy), and, to examine the change in activation across fearful face blocks, fearful block 3 vs. fearful block 1. We added translation and rotation motion regressors to the first-level statistical models to control for movement.

In the second-level group analyses, we ran voxelwise one-group t-tests using the fearful vs. happy contrast, and the fearful block 3 vs. fearful block 1 contrast. We chose to use hypothesis-driven functional regions of interest (ROIs) following earlier studies with this same task (Shin et al., 2005), which examined the brain activation in an independent sample of PTSD participants in response to fearful vs. happy facial expressions. Specifically, we extracted data from the following three functional ROIs in the amygdala (a sphere with a 4 mm radius centered on the following coordinates): *x* = –20, *y* = –8, *z* = –18; *x* = 22, *y* = 4, *z* = –14; *x* = 18, *y* = –6, *z* = –20. We also extracted data from the following four functional ROIs in the rACC/vmPFC (a sphere with a 4 mm radius centered on the following coordinates): *x* = 14, *y* = 48, *z* = 8; *x* = 0, *y* = 46, *z* = –10; *x* = 16, *y* = 38, *z* = 22; *x* = –12, *y* = 52, *z* = −10. For each functional ROI, we extracted BOLD beta values per condition per subject from each region and used this information in correlational analyses to address our *a priori* predictions. *Post hoc* power analyses suggest good power (>0.80) to detect an effect size of 0.61. Based on *z*-scores, none of our variables of interest contained an extreme outlier (>3.29).

In exploratory analyses, we used separate independent-sample *t*-tests to determine whether the extracted ROI data differed between participants who completed vs. dropped out of treatment.

## 3. Results

### 3.1. Clinical change

Participants' demographic and clinical characteristics are presented in Table 1. According to the MINI, our participant sample met criteria for a combination of the following co-occurring diagnoses, major depressive disorder (*n* = 8), major depressive episodes (*n* = 1), panic disorder (PD; *n* = 2), PD with agoraphobia (*n* = 2), PD without agoraphobia (*n* = 2), agoraphobia (*n* = 1), social anxiety disorder (*n* = 1), generalized anxiety disorder (*n* = 4), dysthymia (*n* = 1), and specific phobia (*n* = 2). Paired-sample *t*-tests comparing pre-treatment vs. post-treatment symptom severity scores showed a statistically significant improvement in symptom severity, as measured by both the SPRINT *t*(15) = 4.88, *p* < 0.001 and CGI-S *t*(15) = 5.72, *p* < 0.001. Of the 16 participants who completed treatment, nine participants were considered responders (defined as exhibiting ≥50% improvement on the SPRINT) and seven were considered non-responders (as they experienced < 50% improvement on the SPRINT). Supplementary Table 2 provides the demographic and clinical characteristics of these two groups.

### 3.2. fMRI data

#### 3.2.1. Fearful vs. happy contrast

**Figure 1:**
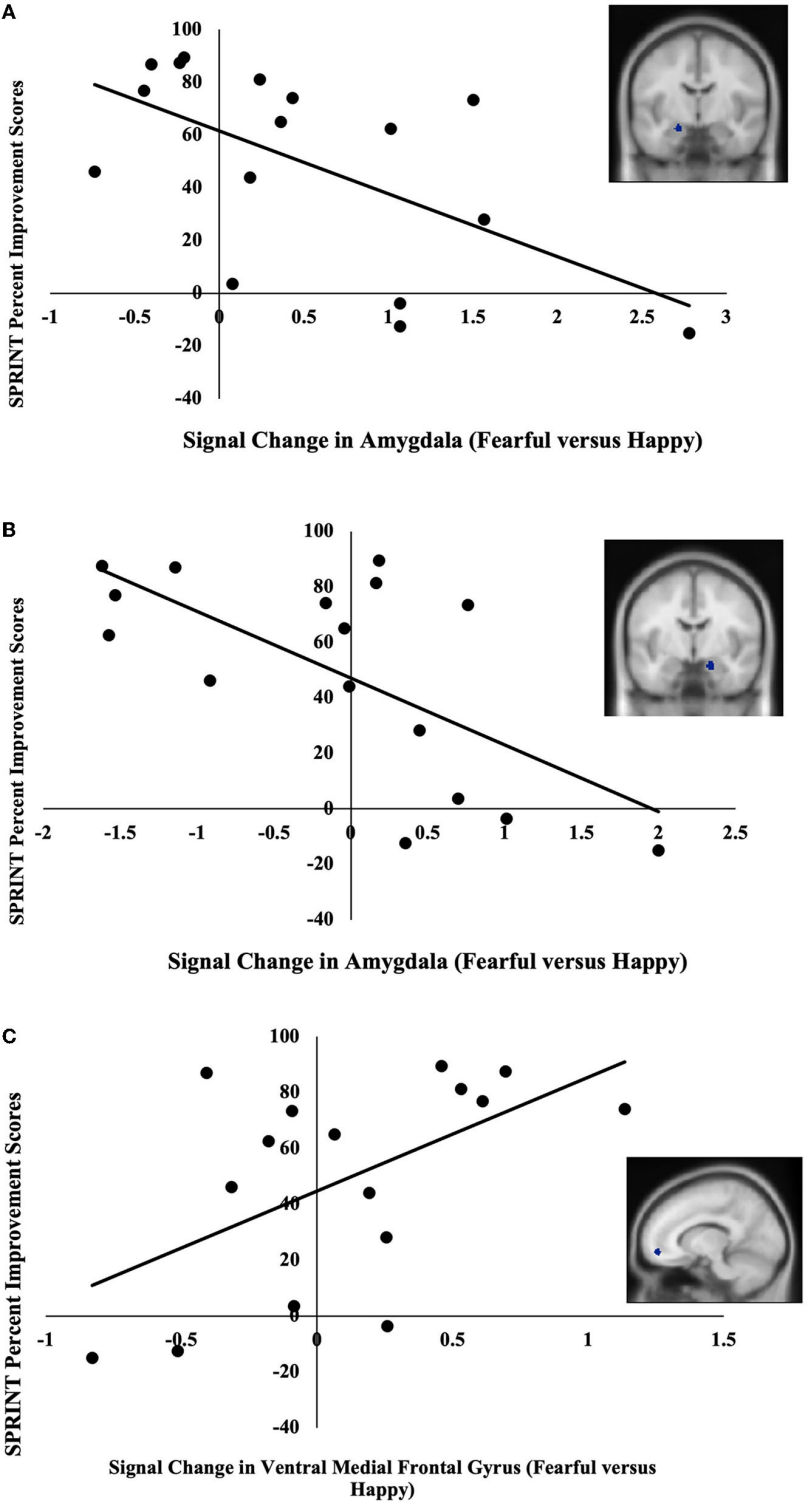
**(A)** Negative correlation between pre-treatment activation (fearful vs. happy) in the left amygdala (MNI coordinates: *x* = –20, *y* = –8, *z* = –18) and SPRINT percent improvement scores. **(B)** Negative correlation between pre-treatment activation (fearful vs. happy) in the right amygdala (*x* = 18, *y* = –6, *z* = –20) and SPRINT percent improvement scores. **(C)** Positive correlation between pre-treatment activation (fearful vs. happy) in ventral medial frontal gyrus (*x* = –12, *y* = 52, *z* = –10) and SPRINT percent improvement scores.

The BOLD signal change in two of the three amygdala ROIs and one of the four rACC/vmPFC ROIs significantly correlated with symptom-related outcome measures. Specifically, BOLD signal changes in the left (MNI; *x* = –20, *y* = –8, *z* = –18) and right (MNI; *x* = 18, *y* = –6, *z* = –20) amygdala negatively correlated with SPRINT percent improvement scores *r*(14) = −0.59, *p* = 0.02 (Figure 1A), *r*(14) = −0.66, *p* = 0.01 (Figure 1B), respectively. These extracted values also negatively correlated with SPRINT change scores when controlling for SPRINT pre-treatment scores, *r*(13) = −0.53, *p* = 0.04, *r*(13) = −0.61, *p* = 0.02, respectively. Furthermore, BOLD signal change in the ventral medial frontal gyrus (MNI; *x* = –12, *y* = 52, *z* = –10) positively correlated with SPRINT percent improvement *r*(14) = 0.55, *p* = 0.03 (Figure 1C) and positively correlated with SPRINT change scores when controlling for SPRINT pre-treatment scores *r*(13) = 0.63, *p* = 0.01. Finally, an extracted BOLD signal change in the right amygdala (MNI; *x* = 18 *y* = –6 *z* = –20) was negatively correlated with CGI-S percent improvement scores *r*(14) = −0.62, *p* = 0.01 and CGI-S change scores when controlling for pre-treatment CGI-S scores *r*(13) = −0.65, *p* = 0.01. Thus, lesser amygdala activation and greater vmPFC activation in response to fearful vs. happy facial expressions were associated with greater improvement following PE. No significant associations were found between pre-treatment symptom severity (SPRINT, CGI-S scores) and our ROI data (*p*s > 0.11).

#### 3.2.2. Fearful block 3 vs. fearful block 1 contrast

**Figure 2:**
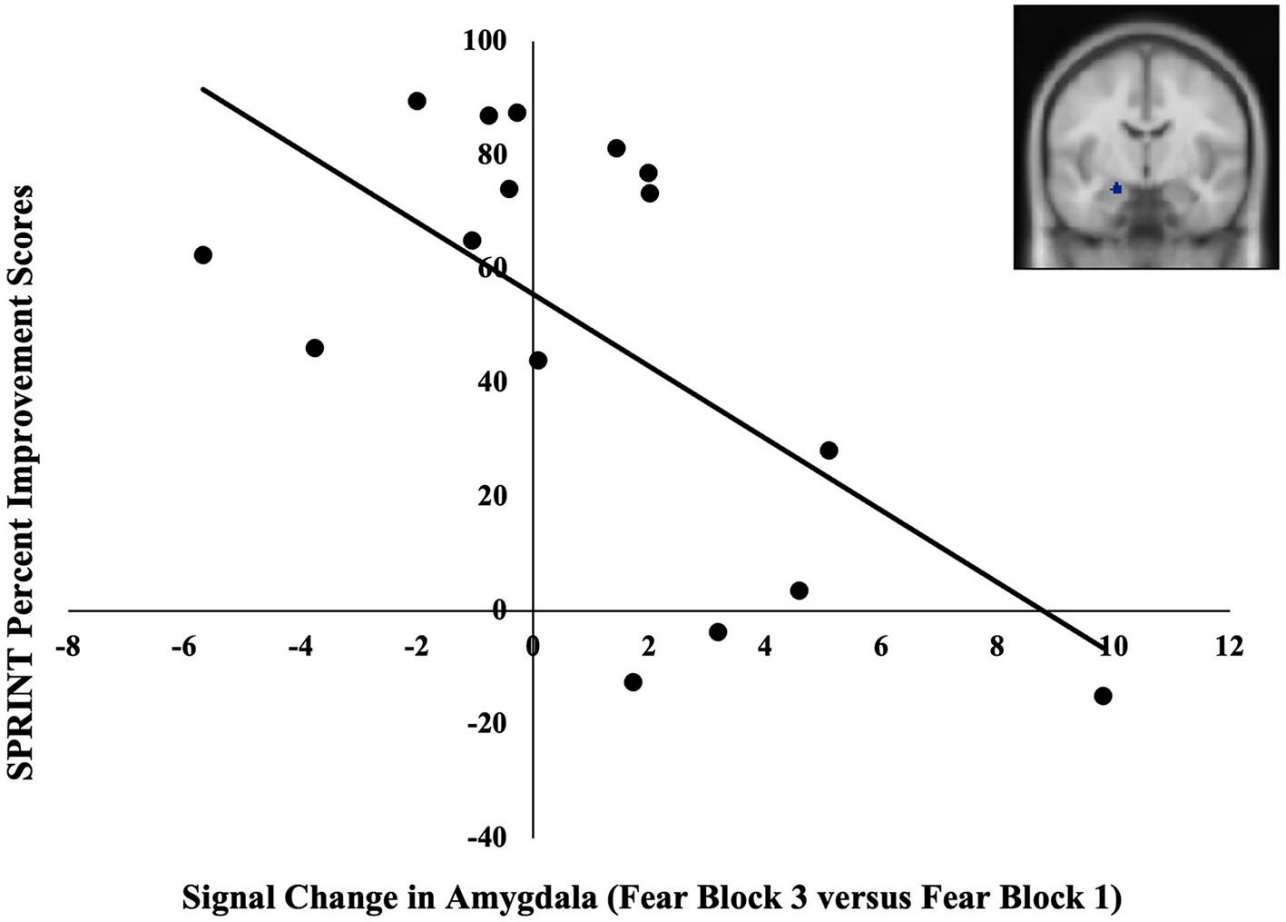
Negative correlation between pre-treatment changes in the BOLD signal in the left amygdala (MNI coordinates: *x* = −20, *y* = –8, z = −18) in late vs. early blocks of fearful facial expressions and SPRINT percent improvement scores. A greater increase in the BOLD signal in the amygdala across blocks of fearful facial expressions was associated with lesser symptomatic improvement.

BOLD signal change in one of the three amygdala ROIs and none of the four rACC/vmPFC ROIs was significantly correlated with symptom-related outcome measures. Specifically, BOLD signal change in the left amygdala (MNI; *x* = –20, *y* = –8, *z* = –18) across blocks of fearful facial expressions was negatively correlated with SPRINT percent improvement scores *r*(14) = −0.62, *p* = 0.01 (Figure 2). These extracted values were also negatively correlated with SPRINT change scores when controlling for SPRINT pre-treatment scores *r*(13) = −0.55, *p* = 0.03. Thus, a greater decline in amygdala responses from the first to the last fearful facial expression block was associated with greater symptomatic improvement. No significant associations were found between pre-treatment symptom severity (SPRINT, CGI-S scores) and our ROI data (*p*s > 0.14).

### 3.3. Exploratory analyses

#### 3.3.1. Valence and arousal ratings

Bivariate Pearson correlations between average participant ratings of the facial expressions and SPRINT percent improvement were not significant (all *p*s > 0.31) (see Table 2 for mean and standard deviation of ratings). Valence ratings of happy faces were significantly positively correlated with CGI-S change scores *r*(14) = 0.53, *p* = 0.03. This correlation remained significant after controlling for CGI-S pre-treatment scores *r*(13) = 0.58, *p* = 0.02. Thus, individuals who rated happy facial expressions as more positive before treatment had better symptom reduction.

**Table 2:** Mean and standard deviations of valence and arousal ratings for each face type of facial expression (fearful, happy, and neutral).

**Face type**	**Valence Mean (SD)**	**Arousal Mean (SD)**
Fearful	−2.11 (0.63)	3.91 (1.45)
Happy	2.23 (0.54)	3.57 (1.29)
Neutral	−0.53 (0.77)	1.93 (1.29)

SD, standard deviation. *n* = 16.

#### 3.3.2. Completers vs. dropouts

These two groups did not differ with respect to BOLD signal change in the fearful vs. happy contrast in any of the amygdala ROIs. However, those who completed the study had a greater BOLD signal change in two of the four rACC/vmPFC ROIs, relative to those who dropped out: rACC (MNI; *x* = 14, *y* = 48, *z* = 8 and MNI; *x* = 0, *y* = 46, *z* = –10), *t*(21) = 2.89, *p* = 0.01, *t*(17.28) = 2.14, *p* = 0.047 (see Supplementary Figures 1, 2). Even when controlling for education (which significantly differed between these two groups), participants who completed the study still exhibited significantly greater brain activation in the two rACC/vmPFC ROIs (MNI; *x* = 14, *y* = 48, *z* = 8), *F*(1,20) = 4.58, *p* = 0.045, (MNI; *x* = 0, *y* = 46, *z* = –10), *F*(1,20) = 4.60, *p* = 0.045. Completed and dropout groups did not show any difference in any other brain response variables (*p*s > 0.10).

## 4. Discussion

In line with our hypotheses, we found that lesser pre-treatment activation in the amygdala and greater pre-treatment activation in the rACC/vmPFC in response to fearful vs. happy facial expressions was each related to greater symptomatic improvement with PE. In addition, we found that a greater decline in amygdala responses from the first to the last fearful facial expression block was also associated with greater improvement.

Our finding of a negative correlation between pre-treatment amygdala activation and symptomatic improvement is consistent with previous findings (van Rooij et al., 2021). Human and animal studies have highlighted the active role the amygdala plays in fear conditioning and extinction learning (Davis, 1992; LeDoux, 1993; Phelps et al., 2004). Overall, studies have found the amygdala to be hyperresponsive in PTSD (reviewed in Kredlow et al., 2022), and this hyperresponsivity likely not only contributes to the development and maintenance of the symptoms of the disorder but also may affect symptom reduction. For PE to be successful, a patient needs to activate their traumatic memory and reappraise that they are now in a safe context, allowing the patient to learn that some associations that were created during the time of the trauma are now erroneous and not generalizable to their current situation (Foa and Meadows, 1997). Our results suggest that excessive pre-treatment amygdala activation could contribute to greater difficulty for some participants to reprocess relevant information and create safety associations during PE therapy. In other words, there may be an upper limit of amygdala activation beyond which PE treatment gains are smaller.

Consistent with prediction, we also found that a decreasing BOLD signal in the amygdala across fearful facial expression blocks was associated with greater improvement. Indeed, amygdala responses typically decline over repeated presentations of emotional facial expression stimuli in healthy participants (Wright et al., 2001; Fischer et al., 2003). We speculate that patients with a greater propensity for amygdala habituation with repeated presentations of fearful face stimuli at baseline may have been better able to experience a reduction in distress during their repeated exposures to PE, hence resulting in their overall greater reduction of symptoms. In other words, neural reactivity to repeated fearful face stimuli showed promise as an individual predictor of responses to extinction-based psychotherapy for PTSD, consistent with the idea that the development of a personalized medicine approach in psychiatry might improve outcomes once refined and adapted in ways it could be feasibly applied in the clinic. Further investigation of amygdala habituation as a potential predictor of responses to treatment seems warranted.

In line with our hypotheses, greater pre-treatment rACC/vmPFC activation was associated with a greater response to PE. The rACC/vmPFC plays a crucial role in the acquisition and retention of extinction, the likely mechanisms underlying PE. We speculate that individuals who have a greater activation in the rACC/vmPFC may have a greater capacity to learn and remember that cues used to associate with threat no longer do. Indeed, previous studies have found similar results in the rACC/vmPFC (Felmingham et al., 2007; Helpman et al., 2016; Zhu et al., 2018; Duval et al., 2020). However, we did not find significant associations between the BOLD signal change in rACC/vmPFC over repeated presentations of fearful faces and symptomatic improvement. Although the reasons for this are unclear, perhaps the functional ROIs in the medial prefrontal cortex that we used, which were taken from a fearful vs. happy contrast in a previous independent cross-sectional study of PTSD, did not sufficiently encompass the brain regions that are associated with symptom reduction. It is also possible that the relationship between a signal change in the rACC/vmPFC over repeated presentations and symptomatic improvement is not linear.

Exploratory analyses revealed two main findings. First, we found that more positive valence ratings of happy facial expressions were associated with greater symptom reduction, although this relationship was significant with only one measure of symptomatic change. Nevertheless, this may be an important finding to replicate in the future as behavioral measures are more cost-efficient and potentially less burdensome on the patient. Second, we discovered differences in pre-treatment brain activation between those who completed PE treatment vs. those who did not. Specifically, those who completed treatment had a greater pre-treatment rACC activation during the fearful vs. happy contrast compared to those who prematurely dropped out of treatment. Given the important role of the rACC/vmPFC in downregulating fear responses in the presence of safety cues, it could be that individuals with greater activation were better able to successfully create safety associations early on in treatment, thereby decreasing distress and increasing the likelihood of completing treatment. However, it is interesting to note that pre-treatment rACC/vmPFC activation was associated with treatment response in only one of the four rACC/vmPFC ROIs across the entire sample. Pre-treatment rACC/vmPFC activation may better predict improvement in specific subtypes of PTSD symptoms, such as avoidance symptoms, rather than total PTSD symptom severity. Indeed, one group found that rACC/vmPFC activation during script-driven imagery (MNI, *x* = –8, *y* = 42, *z* = –4) was negatively associated with avoidance symptoms (Hopper et al., 2007). Although we were unable to break down PTSD symptom total scores into subscores due to missing data, it remains possible that pre-treatment rACC/vmPFC activation may predict improvement in avoidance symptoms per se.

## 5. Limitations and future directions

Our study has several limitations, including a relatively small sample size and a focus on only one type of treatment (PE). Our findings need replication in a larger sample and extension to include other treatments, such as cognitive processing therapy (CPT) or pharmacological interventions, to determine whether amygdala and rACC/vmPFC function predict response to those treatments as well. To date, several studies have found that greater amygdala activation significantly predicts response to treatments including, trauma-focused cognitive behavioral therapy (Cisler et al., 2015; van Rooij et al., 2016; Bryant et al., 2020), eye-movement desensitization and reprocessing therapy (van Rooij et al., 2016), and pharmacological interventions (Sheynin et al., 2020). We suspect that our findings will generalize to other treatment modalities; nevertheless, additional research is needed. In addition, we did not include a waitlist comparison group to control for the potential influence of the passage of time on symptoms. Finally, given the different variables that have been found to predict symptom reduction in PTSD, future larger studies should include machine learning algorithms that identify optimal combinations of clinical, demographic, and neuroimaging data that most accurately predict responses to trauma-focused psychotherapy.

## Data availability statement

The datasets presented in this article are not readily available because the IRB approval for this research did not explicitly allow sharing of the data, which include brain images and clinical treatment outcomes. Requests to access the datasets should be directed to Lisa.Shin@tufts.edu.

## Ethics statement

The studies involving human participants were reviewed and approved by Partners Healthcare System Institutional Review Board. The patients/participants provided their written informed consent to participate in this study.

## Author contributions

SR and LS contributed to the conception and design of the study. LS led data collection, supervised data analysis, and contributed to the writing of the manuscript. CH, MV, and NK contributed to data collection. MC, EB, LM, PS, SR, and NS contributed to participant recruitment and PE. CH performed the statistical analysis with a contribution from JF. CH wrote all drafts of the manuscript. All authors contributed to the manuscript revision and approved the submitted version.

## References

[B1] Aupperle R. L. Allard C. B. Simmons A. N. Flagan T. Thorp S. R. Norman S. B.. (2013). Neural responses during emotional processing before and after cognitive trauma therapy for battered women. Psychiatry Res. 214, 48–55. 10.1016/j.pscychresns.2013.05.001 23916537

[B2] Ball T. M. Stein M. B. Ramsawh H. J. Campbell-Sills L. Paulus M. P. (2014). Single-subject anxiety treatment outcome prediction using functional neuroimaging. Neuropsychopharmacology 39, 1254–1261. 10.1038/npp.2013.328 24270731 PMC3957121

[B3] Bradley R. Greene J. Russ E. Dutra L. Westen D. (2005). A multidimensional meta-analysis of psychotherapy for PTSD. Am. J. Psychiatry 162, 214–227. 10.1176/appi.ajp.162.2.214 15677582

[B4] Breiter H. C. Etcoff N. L. Whalen P. J. Kennedy W. A. Rauch S. L. Buckner R. L.. (1996). Response and habituation of the human amygdala during visual processing of facial expression. Neuron. 17, 875–887. 10.1016/S0896-6273(00)80219-6 8938120

[B5] Bryant R. A. Erlinger M. Felmingham K. Klimova A. Williams L. M. Malhi G.. (2021). Reappraisal-related neural predictors of treatment response to cognitive behavior therapy for post-traumatic stress disorder. Psychol. Med. 51, 2454–2464. 10.1017/S0033291720001129 32366351

[B6] Bryant R. A. Erlinger M. Felmingham K. Malhi G. S. O'Donnell M. L. Williams L. M.. (2020). Differential neural predictors of treatment response for fear and dysphoric features of posttraumatic stress disorder. Depress. Anxiety 37, 1026–1036. 10.1002/da.23061 32579790

[B7] Bryant R. A. Felmingham K. Kemp A. Das P. Hughes G. Peduto A.. (2008). Amygdala and ventral anterior cingulate activation predicts treatment response to cognitive behaviour therapy for post-traumatic stress disorder. Psychol. Med. 38, 555–561. 10.1017/S0033291707002231 18005496

[B8] Cisler J. M. Sigel B. A. Kramer T. L. Smitherman S. Vanderzee K. Pemberton J.. (2015). Amygdala response predicts trajectory of symptom reduction during Trauma-Focused Cognitive-Behavioral Therapy among adolescent girls with PTSD. J. Psychiatr. Res., 71, 33–40. 10.1016/j.jpsychires.2015.09.011 26522869 PMC4826076

[B9] Connor K. M. Davidson J. R. (2001). SPRINT: a brief global assessment of post-traumatic stress disorder. International Clinical Psychopharmacology 16, 279–284. 10.1097/00004850-200109000-00005 11552771

[B10] Davis M. (1992). The role of the amygdala in fear and anxiety. Annu. Rev. Neurosci. 15, 353–375. 10.1146/annurev.ne.15.030192.002033 1575447

[B11] Duval E. R. Sheynin J. King A. P. Phan K. L. Simon N. M. Martis B.. (2020). Neural function during emotion processing and modulation associated with treatment response in a randomized clinical trial for posttraumatic stress disorder. Depress. Anxiety 37, 670–681. 10.1002/da.23022 32306485 PMC8010611

[B12] Ekman P. Friesen W. V. (1976). Pictures of Facial Affect. Palo Alto, CA: Consulting Psychologists Press.

[B13] Etkin A. Maron-Katz A. Wu W. Fonzo G. A. Huemer J. Vértes P. E.. (2019). Using fMRI connectivity to define a treatment-resistant form of post-traumatic stress disorder. Sci. Transl. Med. 11, eaal3236. 10.1126/scitranslmed.aal3236 30944165 PMC6980337

[B14] Falconer E. Allen A. Felmingham K. L. Williams L. M. Bryant R. A. (2013). Inhibitory neural activity predicts response to cognitive-behavioral therapy for posttraumatic stress disorder. J. Clin. Psychiatry 74, 895–901. 10.4088/JCP.12m08020 24107763

[B15] Felmingham K. Kemp A. Williams L. Das P. Hughes G. Peduto A.. (2007). Changes in anterior cingulate and amygdala after cognitive behavior therapy of posttraumatic stress disorder. Psychol. Sci. 18, 127–129. 10.1111/j.1467-9280.2007.01860.x 17425531

[B16] Fischer H. Wright C. I. Whalen P. J. McInerney S. C. Shin L. M. Rauch S. L. (2003). Brain habituation during repeated exposure to fearful and neutral faces: a functional MRI study. Brain Res. Bull. 59, 387–392. 10.1016/S0361-9230(02)00940-1 12507690

[B17] Foa E. B. Hembree E. A. Rothbaum B. O. (2007). Prolonged Exposure Therapy for PTSD: Emotional Processing of Traumatic Experiences, Therapist Guide (Treatments That Work). Oxford: Oxford University Press.

[B18] Foa E. B. Meadows E. A. (1997). Psychosocial treatments for posttraumatic stress disorder: a critical review. Annual Rev. Psychol. 48, 449–480. 10.1146/annurev.psych.48.1.449 9046566

[B19] Fonzo G. A. Goodkind M. S. Oathes D. J. Zaiko Y. V. Harvey M. Peng K. K.. (2017). PTSD psychotherapy outcome predicted by brain activation during emotional reactivity and regulation. Am. J. Psychiatry 174, 1163–1174. 10.1176/appi.ajp.2017.16091072 28715908 PMC5711543

[B20] Guy W. (2000). Clinical Global Impressions Scale (CGI). Virginia: American Psychiatric Association.

[B21] Helpman L. Marin M. F. Papini S. Zhu X. Sullivan G. M. Schneier F.. (2016). Neural changes in extinction recall following prolonged exposure treatment for PTSD: A longitudinal fMRI study. Neuroimage Clin. 12, 715–723. 10.1016/j.nicl.2016.10.007 27761402 PMC5065048

[B22] Hopper J. W. Frewen P. A. van der Kolk B. A. Lanius R. A. (2007). Neural correlates of reexperiencing, avoidance, and dissociation in PTSD: symptom dimensions and emotion dysregulation in responses to script-driven trauma imagery. J. Trauma. Stress 20, 713–725. 10.1002/jts.20284 17955540

[B23] Karatzias A. Power K. McGoldrick T. Brown K. Buchanan R. Sharp D.. (2007). Predicting treatment outcome on three measures for post-traumatic stress disorder. Eur. Arch. Psychiatry Clin. Neurosci. 257, 40–46. 10.1007/s00406-006-0682-2 16915361

[B24] Karlin B. E. Cross G. (2014). From the laboratory to the therapy room: National dissemination and implementation of evidence-based psychotherapies in the US Department of Veterans Affairs Health Care System. Am. Psychol. 69, 19. 10.1037/a0033888 24001035

[B25] Kennis D. M. van Rooij S. J. H. Reijnen A. Geuze D. E. (2017). The predictive value of dorsal cingulate activity and fractional anisotropy on long-term PTSD symptom severity. Depress. Anxiety 34, 410–418. 10.1002/da.22605 28294478

[B26] Kredlow M. A. Fenster R. J. Laurent E. S. Ressler K. J. Phelps E. A. (2022). Prefrontal cortex, amygdala, and threat processing: implications for PTSD. Neuropsychopharmacology 47, 247–259. 10.1038/s41386-021-01155-7 34545196 PMC8617299

[B27] LeDoux J. E. (1993). Emotional memory: in search of systems and synapses. Ann. N. Y. Acad. Sci. 702, 149–157. 10.1111/j.1749-6632.1993.tb17246.x 8109874

[B28] Maguen S. Li Y. Madden E. Seal K. H. Neylan T. C. Patterson O. V.. (2019). Factors associated with completing evidence-based psychotherapy for PTSD among veterans in a national healthcare system. Psychiatry Res. 274, 112–128. 10.1016/j.psychres.2019.02.027 30784780

[B29] Myers K. M. Davis M. (2007). Mechanisms of fear extinction. Mol. Psychiatry 12, 120–150. 10.1038/sj.mp.4001939 17160066

[B30] Nijdam M. J. de Vries G.-J. Gersons B. P. R. Olff M. (2015). Response to psychotherapy for posttraumatic stress disorder: The role of pretreatment verbal memory performance. J. Clin. Psychiatry 76, e1023–e1028. 10.4088/JCP.14m09438 26335088

[B31] Phelps E. A. Delgado M. R. Nearing K. I. LeDoux J. E. (2004). Extinction learning in humans: role of the amygdala and vmPFC. Neuron 43, 897–905. 10.1016/j.neuron.2004.08.042 15363399

[B32] Protopopescu X. Pan H. Tuescher O. Cloitre M. Goldstein M. Engelien W.. (2005). Differential time courses and specificity of amygdala activity in posttraumatic stress disorder subjects and normal control subjects. Biol. Psychiatry 57, 464–473. 10.1016/j.biopsych.2004.12.026 15737660

[B33] Sheehan D. V. Lecrubier Y. Sheehan K. H. Amorim P. Janavs J. Weiller E.. (1998). The Mini-International Neuropsychiatric Interview (M.I.N.I.): the development and validation of a structured diagnostic psychiatric interview for DSM-IV and ICD-10. J. Clin. Psychiatry. 59 (Suppl. 20), 22–33; quiz 34–57. 9881538

[B34] Sheynin J. Duval E. R. King A. P. Angstadt M. Phan K. L. Simon N. M.. (2020). Associations between resting-state functional connectivity and treatment response in a randomized clinical trial for posttraumatic stress disorder. Depress. Anxiety 37, 1037–1046. 10.1002/da.23075 32668087 PMC7722156

[B35] Shin W. Cannistraro W. McMullin M. Macklin L. Cavanagh K. Orr P.. (2005). A functional magnetic resonance imaging study of amygdala and medial prefrontal cortex responses to overtly presented fearful faces in posttraumatic stress disorder. Arch. Gen. Psychiatry 62, 273–281. 10.1001/archpsyc.62.3.273 15753240

[B36] Shiner B. Westgate C. L. Gui J. Cornelius S. Maguen S. E. Watts B. V.. (2020). Measurement strategies for evidence-based psychotherapy for posttraumatic stress disorder delivery: Trends and associations with patient-reported outcomes. Adm. Pol. Ment. Health 47, 451–467. 10.1007/s10488-019-01004-2 31853686 PMC7159996

[B37] Sripada R. K. Ready D. J. Ganoczy D. Astin M. C. Rauch S. A. (2020). When to change the treatment plan: an analysis of diminishing returns in VA patients undergoing prolonged exposure and cognitive processing therapy. Behav. Ther. 51, 85–98. 10.1016/j.beth.2019.05.003 32005342

[B38] Tarrier N. Sommerfield C. Pilgrim H. Faragher B. (2000). Factors associated with outcome of cognitive-behavioural treatment of chronic post-traumatic stress disorder. Behav. Res. Therapy 38, 191–202. 10.1016/S0005-7967(99)00030-3 10661003

[B39] US Department of Veterans Affairs. (2012). VHA Handbook 1160.05: Local Implementation of Evidence-Based Psychotherapies for Mental and Behavioral Health Conditions. Washington, DC: US Department of Veterans Affairs.

[B40] Valenstein-Mah H. Kehle-Forbes S. Nelson D. Danan E. R. Vogt D. Spoont M. (2019). Gender differences in rates and predictors of individual psychotherapy initiation and completion among Veterans Health Administration users recently diagnosed with PTSD. Psychol. Trauma Theory Res. Pract. Pol. 11, 811. 10.1037/tra0000428 30688508 PMC8237797

[B41] van Rooij S. J. H. Geuze E. Kennis M. Rademaker A. R. Vink M. (2015). Neural correlates of inhibition and contextual cue processing related to treatment response in PTSD. Neuropsychopharmacology 40, 667–675. 10.1038/npp.2014.220 25154707 PMC4289955

[B42] van Rooij S. J. H. Kennis M. Vink M. Geuze E. (2016). Predicting treatment outcome in PTSD: a longitudinal functional mri study on trauma-unrelated emotional processing. Neuropsychopharmacology 41, 1156–1165. 10.1038/npp.2015.257 26289143 PMC4748440

[B43] van Rooij S. J. H. Sippel L. M. McDonald W. M. Holtzheimer P. (2021). Defining focal brain stimulation targets for PTSD using neuroimaging. Dep. Anx. 38, 768–785. 10.1002/da.23159 33876868 PMC8526638

[B44] VanElzakker M. B. Dahlgren M. K. Davis F. C. Dubois S. Shin L. M. (2014). From Pavlov to PTSD: the extinction of conditioned fear in rodents, humans, and anxiety disorders. Neurobiol. Learn. Mem. 113, 3–18. 10.1016/j.nlm.2013.11.014 24321650 PMC4156287

[B45] Watkins L. E. Sprang K. R. Rothbaum B. O. (2018). Treating PTSD: a review of evidence-based psychotherapy interventions. Front. Behav. Neurosci. 12, 258. 10.3389/fnbeh.2018.00258 30450043 PMC6224348

[B46] Whalen P. J. Bush G. McNally R. J. Wilhelm S. McInerney S. C. Jenike M. A.. (1998). The emotional counting Stroop paradigm: a functional magnetic resonance imaging probe of the anterior cingulate affective division. Biol. Psychiatry 44, 1219–1228. 10.1016/S0006-3223(98)00251-0 9861465

[B47] Wright C. I. Fischer H. Whalen P. J. McInerney S. C. Shin L. M. Rauch S. L. (2001). Differential prefrontal cortex and amygdala habituation to repeatedly presented emotional stimuli. Neuroreport 12, 379–383. 10.1097/00001756-200102120-00039 11209954

[B48] Zhu X. Suarez-Jimenez B. Lazarov A. Helpman L. Papini S. Lowell A.. (2018). Exposure-based therapy changes amygdala and hippocampus resting-state functional connectivity in patients with posttraumatic stress disorder. Depress. Anxiety 35, 974–984. 10.1002/da.22816 30260530 PMC6168398

