# Supplementary material for: Pre-treatment amygdala activation and habituation predict symptom change in post-traumatic stress disorder
Source: Front Behav Neurosci. 2023 Jul 10;17:1198244. doi: 10.3389/fnbeh.2023.1198244 (PMC10363634; doi:10.3389/fnbeh.2023.1198244)
Supplement: Supplementary file 1 [file Data_Sheet_1.docx]

**Supplementary Materials**

1. **Results**
   1. **Responders versus non-responders**

BOLD signal change in the right amygdala (MNI; x=18 y=-6 z=-20) significantly differed between responders and non-responders. Specifically, non-responders had significantly greater right amygdala activation compared to responders in the fearful versus happy facial expression contrast, *t*(14)=-2.32, *p*=.04 (see **Supplementary Table 2**). When controlling for age (which significantly differed between groups), the difference between responders versus non-responders in right amygdala (MNI; x=18 y=-6 z=-20) remained significant, *p*=.048. Responder vs. non-responder groups did not differ on any other brain response variable (*p*s>.07).

**Supplementary Table 1**

*Participant Characteristics of Completers and Dropouts*

|  | **Completers (n=16)** | | | **Dropouts (n=7)** | |  |  |
| --- | --- | --- | --- | --- | --- | --- | --- |
|  | **Mean** | **SD** | **Mean** | | **SD** | ***t*** | ***p*** |
| **Age** | 43.62 | 12.57 | 39.29 | | 6.82 | 0.85 | .40 |
| **Education (years)** | 16.25 | 2.59 | 13.79 | | 1.68 | 2.95 | .03* |
| **Time since trauma (years)^a,b^** | 7.60 | 8.97 | 3.13 | | 0.18 | .68 | .51 |
| **SPRINT pre-treatment scores^c^** | 21.38 | 5.76 | 21.60 | | 3.29 | -0.08 | .94 |
| **CGI-S pre-treatment scores^c^** | 4.75 | .77 | 4.60 | | 0.55 | 0.40 | .69 |
| **Fearful versus happy contrast** |  |  |  | |  |  |  |
| **Amygdala** |  |  |  | |  |  |  |
| **-20, -8, -18** | .52 | .93 | -.08 | | 1.13 | 1.34 | .20 |
| **22, 4, -14** | -.12 | 1.21 | -.78 | | 1.37 | 1.17 | .26 |
| **18, -6, -20** | -.09 | 1.03 | -.12 | | .59 | .07 | .95 |
| **rACC/vmPFC** |  |  |  | |  |  |  |
| **14, 48, 8** | .07 | .29 | -.35 | | .37 | 2.89 | .01** |
| **0, 46, -10** | .20 | 1.10 | -.41 | | .21 | 2.14 | .05* |
| **16, 38, 22** | -.03 | .46 | .17 | | .42 | -1.00 | .33 |
| **-12, 52, -10** | .11 | .51 | -.08 | | .41 | .87 | .40 |
| **Fear block 3 versus fear block 1 contrast** |  |  |  | |  |  |  |
| **Amygdala** |  |  |  | |  |  |  |
| **-20, -8, -18** | 1.00 | 3.68 | 1.69 | | 5.34 | -.36 | .72 |
| **22, 4, -14** | 1.44 | 6.25 | 2.06 | | 5.90 | -.22 | .83 |
| **18, -6, -20** | 3.20 | 3.82 | .52 | | 3.24 | 1.62 | .12 |
| **rACC/vmPFC** |  |  |  | |  |  |  |
| **14, 48, 8** | .14 | 1.29 | -.17 | | 2.16 | .43 | .67 |
| **0, 46, -10** | -.42 | 4.09 | -.66 | | 2.25 | .15 | .88 |
| **16, 38, 22** | -.49 | 1.82 | .31 | | 1.72 | -.99 | .33 |
| **-12, 52, -10** | -.91 | 3.01 | -1.24 | | 1.91 | .27 | .79 |

*Notes.*

SPRINT = Short Posttraumatic Stress Disorder Rated Interview

CGI-S = Clinical Global Improvement Severity Scale

rACC = rostral anterior cingulate cortex

vmPFC = ventromedial prefrontal cortex

SD = Standard deviation

** = *p*=.01

* = *p*=.05

^a^Completers (*n*=13)

^b^Dropouts (*n*=2)

^c^Completers (*n*=16), dropouts (*n*=5)

**Supplementary Table 2**

*Participant Characteristics of Responders and Non-Responders*

|  | **Responders (n=9)** | | **Non-Responders (n=7)** | |  |  |
| --- | --- | --- | --- | --- | --- | --- |
|  | **Mean** | **SD** | **Mean** | **SD** | ***t*** | ***p*** |
| **Age** | 38.11 | 13.09 | 50.71 | 7.91 | -2.24 | .04* |
| **Education (years)** | 16.67 | 2.60 | 15.71 | 2.69 | .72 | .49 |
| **Time since trauma (years)^a^** | 9.64 | 10.47 | 4.33 | 5.29 | 1.04 | .32 |
| **SPRINT pre-treatment scores** | 19.22 | 4.68 | 24.14 | 6.15 | -1.82 | .09 |
| **SPRINT post-treatment scores** | 4.22 | 1.92 | 21.29 | 7.89 | -5.59 | .001*** |
| **SPRINT changes scores** | 15.00 | 4.53 | 2.86 | 5.79 | 4.72 | <.001*** |
| **SPRINT percent improvement scores** | 77.44 | 9.73 | 12.95 | 26.11 | 6.21 | <.001*** |
| **CGI-S pre-treatment scores** | 4.56 | .73 | 5.00 | .82 | -1.15 | .27 |
| **CGI-S post-treatment scores** | 2.33 | .50 | 4.28 | 1.25 | -3.89 | .01** |
| **CGI-S change scores** | 2.22 | .97 | .71 | .49 | 3.74 | .002 |
| **CGI-S percent improvement scores** | 47.41 | 14.86 | 15.71 | 10.97 | 4.72 | <.001*** |
| **CGI-I Scores** | 1.78 | .67 | 3.29 | 1.11 | -3.38 | .005** |
| **Fearful versus happy contrast** |  |  |  |  |  |  |
| **Amygdala** |  |  |  |  |  |  |
| **-20, -8, -18** | .25 | .66 | .86 | 1.15 | -1.33 | .20 |
| **22, 4, -14** | -.43 | 1.54 | .28 | .40 | -1.33 | .21 |
| **18, -6, -20** | -.55 | .91 | .51 | .90 | -2.32 | .04* |
| **rACC/vmPFC** |  |  |  |  |  |  |
| **14, 48, 8** | .00 | .27 | .14 | .31 | -.94 | .36 |
| **0, 46, -10** | .44 | .91 | -.10 | 1.31 | .98 | .34 |
| **16, 38, 22** | .10 | .57 | -.20 | .16 | 1.38 | .19 |
| **-12, 52, -10** | .31 | .49 | -.15 | .42 | 1.96 | .07 |
| **Fear block 3 versus fear block 1 contrast** |  |  |  |  |  |  |
| **Amygdala** |  |  |  |  |  |  |
| **-20, -8, -18** | -.53 | 2.39 | 2.96 | 4.26 | -2.08 | .06 |
| **22, 4, -14** | 3.96 | 6.25 | -1.80 | 4.86 | 2.01 | .06 |
| **18, -6, -20** | 3.30 | 3.52 | 3.07 | 4.47 | .11 | .91 |
| **rACC/vmPFC** |  |  |  |  |  |  |
| **14, 48, 8** | .04 | 1.21 | .27 | 1.49 | -.33 | .74 |
| **0, 46, -10** | -1.74 | 3.71 | 1.28 | 4.18 | -1.53 | .15 |
| **16, 38, 22** | -.19 | 2.26 | -.88 | 1.08 | .74 | .47 |
| **-12, 52, -10** | -.73 | 1.76 | -1.13 | 4.29 | .25 | .80 |

*Notes.* Responder is defined here as a patient who had ≥ 50% reduction in symptoms at the end of treatment.

SPRINT = Short Posttraumatic Stress Disorder Rated Interview

CGI-S = Clinical Global Improvement Severity Scale

rACC = rostral anterior cingulate cortex

vmPFC = ventromedial prefrontal cortex

SD = Standard deviation

*** = *p*<.001

** = *p*<.01

* = *p*<.05

^a^Responders (*n*=8), non-responders (*n*=5)


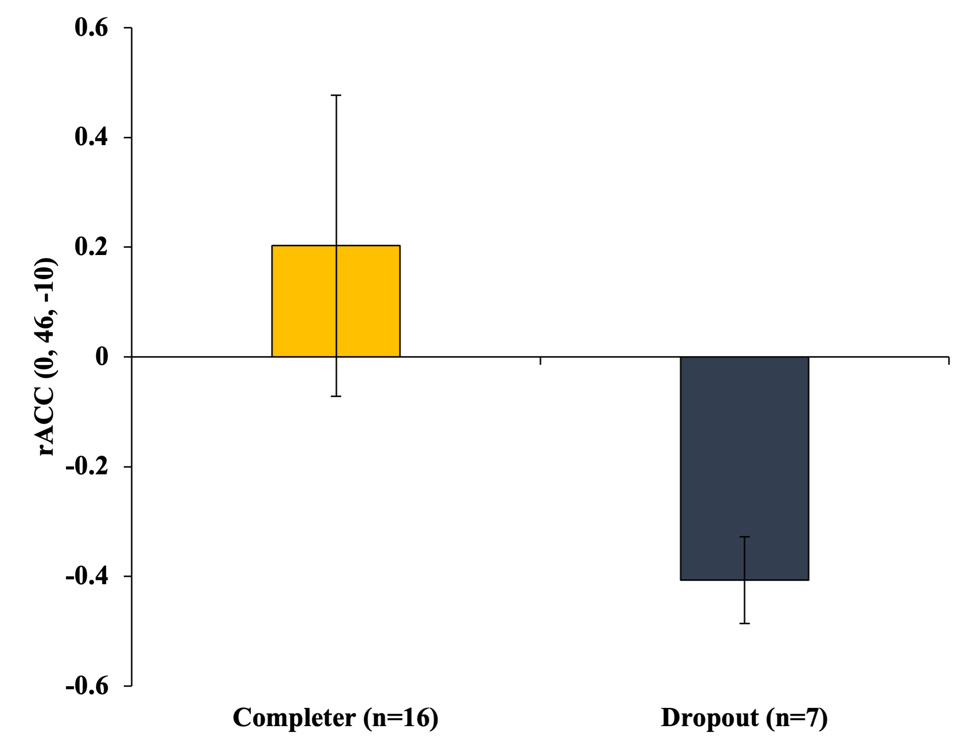


**Supplementary Figure 1.** Differences in the rostral anterior cingulate cortex (rACC; MNI x=0 y=46 z=-10) activation (fearful versus happy) between those who completed treatment (*n*=16) and those who prematurely dropped out of treatment (*n*=7). Error bars represent standard error.


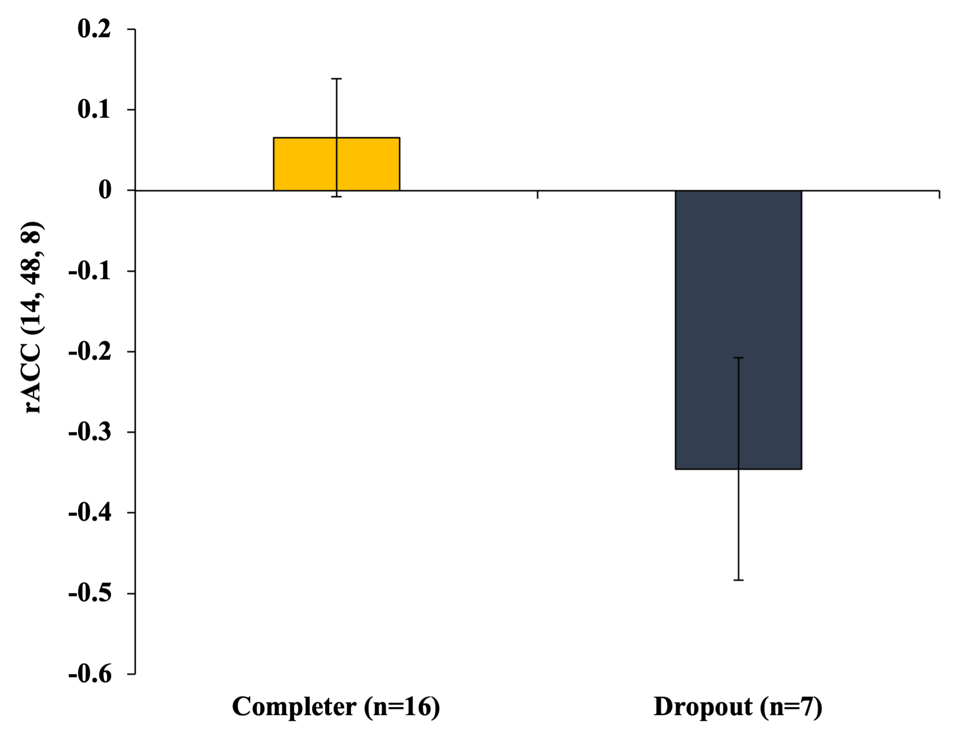


**Supplementary Figure 2.** Differences in the rostral anterior cingulate cortex (rACC; MNI x=14 y=48 z=8) activation (fearful versus happy) between those who completed treatment (*n*=16) and those who prematurely dropped out of treatment (*n*=7). Error bars represent standard error.
